# Domain-Specific and Total Sedentary Behavior Associated with Gait Velocity in Older Adults: The Mediating Role of Physical Fitness

**DOI:** 10.3390/ijerph17020593

**Published:** 2020-01-16

**Authors:** Mario Kasović, Lovro Štefan, Martin Zvonař

**Affiliations:** 1 Faculty of Kinesiology, Department of General and Applied Kinesiology, University of Zagreb, 10000 Zagreb, Croatia; mario.kasovic@kif.hr; 2 Faculty of Sports Studies, Masaryk University, 60177 Brno, Czech Republic; zvonar@fsps.muni.cz

**Keywords:** screen-time, walking speed, performance, aged

## Abstract

Although it has been well-documented that older adults spend a significant amount of time being sedentary and have slower gait velocity, little is known of how physical fitness mediates the association between them. The main purpose of this study was to explore whether objectively measured physical fitness mediates the association between domain-specific and total sedentary behavior and gait velocity. We recruited 120 older adults aged ≥ 60 years. Sedentary behavior was assessed by the Measure of Older Adults’ Sedentary Time questionnaire. We used a Zebris pressure platform to assess gait velocity. To assess the level of overall physical fitness, we summed the *z*-scores of seven tests: (1) waist circumference, (2) chair stand in 30 s, (3) arm curl in 30 s, (4) 2-min step test, (5) chair sit-and-reach test, (6) back scratch test, and (7) 8-foot up-and-go test. Overall physical fitness was obtained by summing up all physical test *z*-scores. Gait velocity was significantly associated with all domain-specific and total sedentary behavior (*β* = −0.04 to −0.35, *p* < 0.05). Overall physical fitness was significantly associated with all domain-specific and total sedentary behavior (*β* = −0.21 to −1.24, *p* < 0.001) and gait velocity (*β* = 0.23 to 0.24, *p* < 0.001). When physical fitness was put as the mediator, significant direct effects between sedentary behavior and gait velocity disappeared. Results indicate that physical fitness fully mediates the association between sedentary behavior and gait velocity in older adults.

## 1. Introduction

In the last 50 years, the population of people aged 60 years and older has increased by 2%, with estimation that the number will increase to 22% by 2050 [1]. Older adults are facing many health-related consequences, including twice as many disabilities and four times as many physical limitations as people who are aged less than 60 years [2]. Those issues are primarily evident in individuals with poor physical performance and more time spent in sedentary behaviors. Sedentary behaviors are defined as “any waking behavior characterized by energy expenditure ≤ 1.5 metabolic equivalents, while in a sitting, reclining or lying posture” [3]. Specifically, older adults spend more than 4 h/day sedentary. While observing domain-specific sedentary behaviors, 65% of them spend more than 3 h/day in front of a screen, over 55% report watching television for more than 2 h/day [4], and over 3 h/day in leisure-time sedentary behavior [5]. Independent of physical activity, sedentary behaviors have often been associated with negative health outcomes, including overweight/obesity status, elevated blood pressure and total cholesterol, and lower levels of self-esteem, physical fitness, and academic achievement [6].

Despite the well-documented health benefits of physical activity, walking is the most common weight-bearing physical activity in older adults [7]. Therefore, the structure and function of the foot represent the main factors of normal gait. Accumulating evidence has shown that older adults, compared to younger populations, have somewhat different foot structure and function, including flatter feet, intrinsic foot muscle weakness, altered plantar pressure loading patterns during walking, and reduced plantar tactile sensitivity [8], which in addition may lead to impaired gait velocity. Moreover, previous studies have shown that attention and executive functions, which are described as a set of higher cognitive processes that modulate behavior, significantly decline with age, leading to slower gait velocity and higher risk of falls [9]. Preferred gait velocity has been associated with positive health effects, including lower risk of falls and reduced risk of all-cause mortality [10]. However, the association between sedentary behavior and gait velocity has been less studied [11,12]. Slow gait velocity has been identified as a significant predictor of cessation of regular physical activity [11] and more time spent in sedentary behavior [12]. Thus, slow gait velocity may potentially discourage old people to engage in leisure-time physical activity and lead to a more sedentary lifestyle [12].

The key of successful aging represents functional independence and maintaining high quality of life [13], often accompanied by a high level of physical fitness. In older adults, the level of physical fitness is significantly reduced due to higher rates of disabilities and limitations [2]. Although a higher level of physical fitness reduces the time spent in sedentary behavior [14,15] and increases gait velocity [13,16,17,18], according to available literature no study has investigated the potential mediating role of physical fitness in the association between domain-specific and total sedentary behavior and gait velocity. Since only 25% of older adults meet the recommended levels of physical activity proposed by the World Health Organization [19], it is necessary to identify individuals in need of increasing their physical fitness level in order to decrease sedentary lifestyle and improve gait parameters.

Therefore, the main purpose of this study was to explore whether objectively measured physical fitness mediates the association between domain-specific and total sedentary behavior and gait velocity.

## 2. Materials and Methods

### 2.1. Study Participants

In this cross-sectional study, we recruited older adults aged ≥60 years from five neighborhoods in the city of Zagreb. In the first stage, we spread information about the main aims and benefits of the study via posters. Of approximately 1500 adults aged ≥60 years living in five neighborhoods, the estimated sample size for the confidence level of 95% and confidence interval (margin of error) of 10% was 110. In order to correct for possible missing data, we recruited 210 participants, of which 73 did not provide full data and 17 could no longer be in the study due to personal issues. Finally, we based our study on 120 older women (100%). Based on previous studies [20], the inclusion criteria were: (1) being ≥60 years old, (2) living independently in the community, (3) passing the Short Portable Mental Status Questionnaire [21], (4) being able to ambulate for at least 10 m with or without an aid, (5) being free from neurological diseases, and (6) could arrange their own transport to a testing venue in their community. All participants gave written informed consent before entering the study. All procedures performed in this study were anonymous and according to Declaration of Helsinki, and were approved by the Faculty of Kinesiology, University of Zagreb, Croatia (Ethics code number: 2019).

### 2.2. Gait Velocity Assessment

To assess the level of plantar pressure under each participant’s feet while walking, we used a Zebris plantar pressure platform (FDM; GmbH, Munich, Germany; number of sensors: 11.264; sampling rate: 100 Hz; sensor area: 149 cm × 54.2 cm). According to previous studies, the calibrated platform was placed on a firm, level surface, with a custom-designed dense walkway surrounding the plate to provide a level walking surface [20]. Each participant was instructed to walk at a comfortable speed across the platform without shoes and socks. Additionally, all participants were required to look straight forward, not targeting the pressure platform. When they reached the end of the walkway, they needed to turn 180° around and continue to walk again over the platform. Finally, when they reached the end of the second walkway (trial), they again turned 180° around and walked a final time across the platform until the end of the walkway. Previous evidence has suggested that collecting 3–5 trials across the pressure platform is more reliable in populations affected with diseases such as arthritis [22]. If we noticed that the participant had targeted the pressure platform or had obvious gait deviations, trials were discarded and we repeated the measurement. Zebris software generated the data regarding the gait velocity in km/h.

### 2.3. Sedentary Behavior Assessment

To assess sedentary behavior, we used the Measure of Older Adults’ Sedentary Time questionnaire [23]. This questionnaire assesses time spent sitting by covering several sedentary behavior domains: (i) watching television, (ii) using a computer/tablet, (iii) reading, (iv) socializing, (v) transportation, and (vi) hobbies/other. This questionnaire has been shown to have good reliability (0.52) and modest validity (0.30), and it is suitable for use in interventions with older adults [23]. For the purpose of this study, we created domain-specific categories of sedentary behavior as done in previous studies [24] as follows: (1) screen-time (watching television and using a computer/tablet), (2) leisure-time (reading, socializing, and hobbies), (3) transport (transport), and (4) total sedentary time (sum of all domain-specific categories).

### 2.4. Physical Fitness Assessment

The Senior Fitness Test was used to assess the level of physical fitness [25]. It is composed of six tests as follows: (1) chair stand in 30 s, (2) arm curl in 30 s, (3) 2-min step test, (4) chair sit-and-reach test, (5) back scratch test, and (6) 8-foot up-and-go test. In addition, we measured waist circumference between the last rib and umbilicus and entered it in the model. Chair stand in 30 s was used to assess lower body strength, and participants needed to come to a full stand from a seated position with arms folded across the chest. Arm curl in 30 s was the second test, representing a general measure of upper-body strength, and involved counting the number of times a person could curl a hand weight (5 pounds or 2.3 kg for women and 8 pounds or 3.6 kg for men) through a full range of motion. The third test included a person stepping in place and raising their knees to a height halfway between the patella (knee cap) and iliac crest (front hip bone). This test is a measure of aerobic endurance, and the results were expressed in the number of steps taken during the two minutes. Next, the chair sit-and-reach test aimed to assess lower-body flexibility. The test involved sitting at the front edge of a stable chair with one leg extended and the other foot flat on the floor. With hands on top of each other and arms outstretched, the participant reached as far forward as possible toward the toes. The score was expressed in cm (a higher score was better) and was measured three times, where the best score was taken in the model. The purpose of the back-scratch test was to assess upper-body flexibility, particularly shoulder flexibility. The test involved reaching one hand over the shoulder and down the back as far as possible and the other hand around the waist and up the middle of the back as far as possible, trying to bring the fingers of both hands together. The score was expressed in cm (a higher score was better) and was measured three times, where the best score was taken in the model. Finally, the 8-foot up-and-go test was used to assess agility and dynamic balance. The test involved getting up from a seated position and walking as quickly as possible around a cone that is 8 feet (2.4 m) away and returning to the seated position. The test was performed two times and the results were expressed in seconds. In addition, we objectively measured height and weight (using a Seca portable stadiometer and scale) and asked the participants about their chronological age.

### 2.5. Data Analysis

Basic descriptive statistics are presented as mean ± SD or median (25th–75th percentile range) for normally and non-normally distributed variables. We calculated *z*-scores for each physical fitness test. To get an overall physical fitness score, we summed all *z*-scores and put this variable into the model. The *z*-score was calculated by subtracting participant’s score in the test with the overall mean score of the whole sample and dividing it by the standard deviation. The *z*-score tells us how many standard deviations an individual score is from the mean. To test whether physical fitness mediated the association between domain-specific and total sedentary behavior and gait velocity, we used a bootstrapping method [26]. Bootstrapping is a nonparametric approach to effect-size estimation and hypothesis testing that makes no assumptions about the shape of the distributions of the variables or the sampling distribution of the statistic. It also produces a test that is not based on large-sample theory, meaning it can be applied to small samples with more confidence [26]. To test for mediation, several criteria needed to be met: (1) a significant regression path (*a*) between the independent variable (domain-specific and total sedentary behavior) and the mediator (physical fitness), (2) a significant regression path (*b*) between the mediator (physical fitness) and the dependent variable (gait velocity), and (3) a significant regression path (*c*) between the independent (domain-specific and total sedentary behavior) and the dependent variable (gait velocity). If the *c* path between the independent and the dependent variables is no longer significant when the mediator is put into the model, one can conclude that the mediator fully mediates the association. Otherwise the mediator only partially mediates the association. In the present study, the 95% confidence interval of the indirect effect was obtained with a 5000 bootstraps resample. All analyses were performed in Statistical Packages for Social Sciences version 23. (SPSS Inc., Chicago, IL, USA) with statistical significance of *p* ≤ 0.05.

## 3. Results

**Figure 1:**
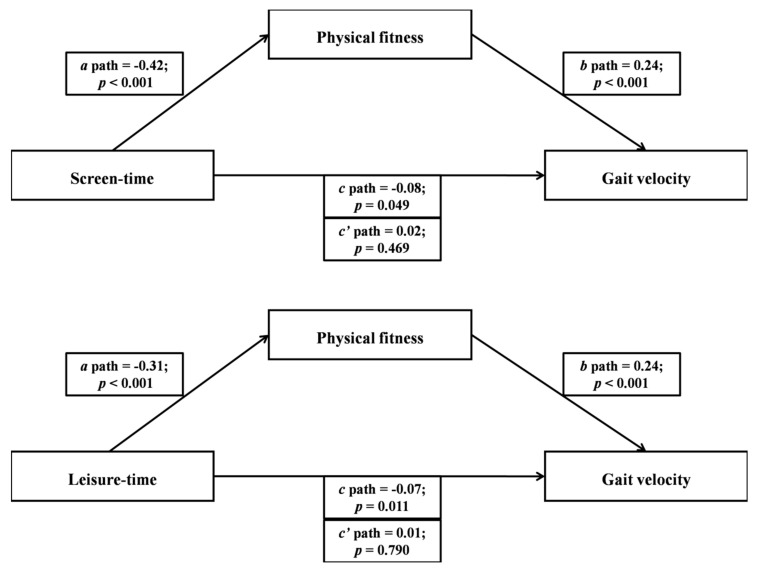
The mediating role of physical fitness in the association between screen-time and leisure-time sedentary behavior with gait velocity (*N* = 120).

**Figure 2:**
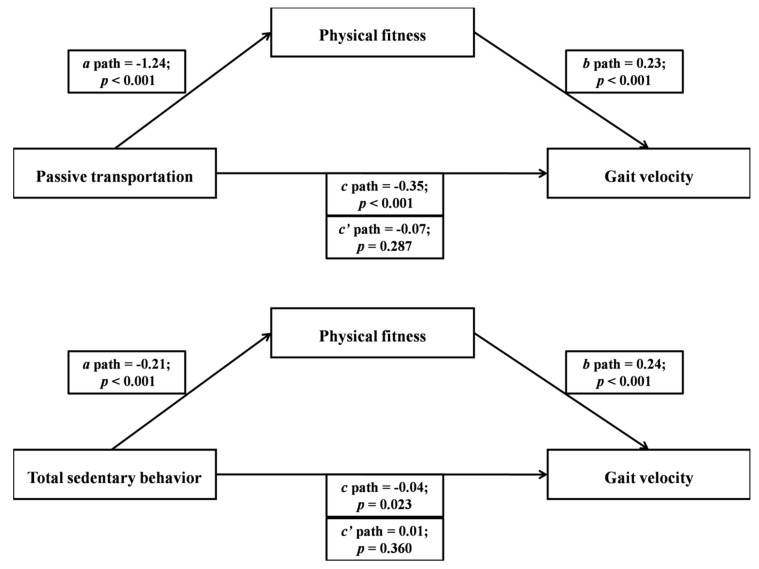
The mediating role of physical fitness in the association between passive transportation and total sedentary behavior with gait velocity (*N* = 120).

Basic descriptive statistics of the study participants are presented in Table 1. Figure 1 and Figure 2 show the mediating role of physical fitness in the association between domain-specific and total sedentary behavior and gait velocity. Multiple regression analyses were conducted to assess each component of the proposed mediation. In Figure 1, screen-time and leisure-time sedentary behaviors were significantly and inversely associated with physical fitness. Second, physical fitness was significantly and positively associated with gait velocity in both screen-time and leisure-time sedentary behavior models. Lastly, results indicated that gait velocity was significantly and inversely associated with screen-time and leisure-time sedentary behaviors. When physical fitness was entered as the mediator, the significant associations between screen-time and leisure-time sedentary behaviors and gait velocity became non-significant. In Figure 2, passive transportation and total sedentary behavior were significantly and inversely associated with physical fitness. Second, physical fitness was significantly and positively associated with gait velocity in both passive transportation and total sedentary behavior models. Lastly, results indicated that gait velocity was significantly and inversely associated with passive transportation and total sedentary behavior. When physical fitness was entered as the mediator, the significant associations between passive transportation and total sedentary behavior and gait velocity became non-significant.

## 4. Discussion

The main purpose of this study was to explore whether objectively measured physical fitness mediates in the association between domain-specific and total sedentary behavior and gait velocity. This is the first study examining the aforementioned purpose. Our results showed that: (1) more time spent in domain-specific and total sedentary behavior was associated with lower physical fitness; (2) higher physical fitness was associated with faster gait velocity; (3) faster gait velocity was associated with less time spent in domain-specific and total sedentary behavior; and (4) physical fitness fully mediated the association between domain-specific and total sedentary behavior and gait velocity.

Our findings that a higher level of physical fitness is associated with faster gait velocity are in line with previous evidence [13,16,17,18]. In cross-sectional studies, walking speed has been associated with knee extensor strength over the entire range of strength [16]. Additionally, gait time decreases linearly with increasing knee extensor strength [17] and strength measure (composed of sum of knee extension, knee flexion, and ankle dorsiflexion muscle strength scores) is the strongest predictor of six-meter walking speed [18]. Moreover, one previous study has shown that hand grip strength was significantly associated with gait stability [13]. Recently, two longitudinal studies examined the association between gait velocity and physical performance [27,28]. Results from those studies indicate that gait speed and physical performance independently predict the risk of all-cause mortality [27]; therefore, both gait velocity and physical fitness serve as significant factors to determine the level of successful aging. Another longitudinal study has shown that slow gait was associated with poor physical function, concluding that gait velocity should be a summary index of lifelong aging and could be a potential screening tool for physical and functional decline [28].

In line with previous studies [14,15,29,30,31,32,33,34] our study shows that the time spent in domain-specific and total sedentary behavior is associated with physical fitness. Sedentary behaviors are considered a new risk factor for health in older adults, regardless of physical performance [29]. Most recently, a study by Sagarra-Romero et al. [30] has shown that sitting for longer than 4 h/d is associated with lower strength, flexibility, and aerobic endurance in older men and balance, strength, agility, walking speed, and aerobic endurance in older women. In general, physical fitness decreases and time spent in sedentary behaviors increases with age, pointing out that special policies and strategies that reduce the time being sedentary and increase physical fitness are, in fact, relevant [35].

Previous evidence has shown that faster gait velocity [13], less time spent in sedentary behaviors [30], and higher level of physical fitness [30] are crucial factors for maintaining successful and healthy aging and higher quality of life [13]. As mentioned in the Introduction section, only 25% of older adults meet the minimum recommended activity levels of at least 150 min of moderate-intensity aerobic physical activity throughout the week or do at least 75 min of vigorous-intensity aerobic physical activity throughout the week, or an equivalent combination of both [19]. Thus, by screening and detecting older adults with poor physical fitness, health professionals could encourage those individuals who are at extreme risk to engage in organized physical activity in order to be less sedentary and improve biomechanical gait parameters.

This study has a few limitations. First, by using a cross-sectional design, we cannot conclude the causality of the mediation. Second, we based our findings on a relatively small sample of participants (*N* = 120), and a larger sample size may have provided a somewhat different strength of the association. Third, we based our study on a sample living in the urban part of the country, speaking Croatian and of only White race. Fourth, to assess the level of sedentary behavior, we used a self-reported measure, which may have under- or over-estimated the time spent in a specific sedentary behavior domain. Future studies should explore longitudinal associations between gait velocity and domain-specific and total sedentary behavior and investigate the mediating role of physical fitness in population-based studies and in different world regions to generate relevant and comparable data.

## 5. Conclusions

In conclusion, this is the first study showing that objectively measured physical fitness fully mediates the association between domain-specific and total sedentary behavior and gait velocity in a sample of older adults aged ≥ 60 years. Therefore, physical fitness assessment should be a significant tool in identifying the risk group of older adults, and interventions aiming to enhance their quality of life and functional independence by using physical fitness must be implemented within communities.

## Figures and Tables

**Table 1:** Basic descriptive statistics of the study participants (*N* = 120).

Study Variables	Mean ± SD
Age (years)	71.01 ± 6.77
Height (cm)	158.92 ± 21.41
Weight (kg)	70.29 ± 12.97
Gait velocity (km/h)	3.00 ± 1.00
Waist circumference (cm)	91 ± 12
Chair stand in 30 s (#)	17 ± 4
Arm curl in 30 s (#)	19 ± 5
2-min step test (#)	170 ± 44
Chair sit-and-reach test (cm) ^†^	7 (1 to 11)
Back scratch test (cm) ^†^	0.8 (−8 to 4)
8-foot up-and-go test (s)	5 ± 1
Overall physical fitness (*z*-score) ^†^	−1 (−2 to 1)
Screen-time (h/d) ^†^	2.18 (0.70 to 4.00)
Leisure-time (h/d) ^†^	3.56 (1.87 to 6.08)
Passive transportation (h/d) ^†^	0.57 (0.14 to 1.14)
Total sedentary behavior (h/d) ^†^	6.83 (3.57 to 10.31)

^†^ denotes using median (25th–75th percentile range); # this means the number of repetitions.
